# Switchable linear to circular polarization conversion in reflection and transmission modes based on vanadium-dioxide

**DOI:** 10.1038/s41598-025-11988-7

**Published:** 2025-08-06

**Authors:** Eman M. Eldesouki, Ahmed Elsayed Abouelez

**Affiliations:** Microwave Engineering Department, Electronics Research Institute (ERI), Cairo, Egypt

**Keywords:** Terahertz, Metamaterials, Polarization converter, Vanadium dioxide, Dual mode, Engineering, Optics and photonics

## Abstract

A design of a switchable dual-mode linear-to-circular polarization converter (LTC-PC) in the terahertz (THz) band is reported based on vanadium dioxide (VO_2_). Adjusting the VO_2_ state allows the converter to alternate between the transmission and reflection modes. In the insulating state, VO_2_ enables transmission mode operation for a forward x- or y-polarized wave. LTC polarization conversion occurs within the frequency bands of 1.26–1.47 THz and 1.83–1.85 THz. Moreover, this mode yields an LTC polarization conversion at a frequency of 1.7 THz. The polarizer operates in reflection mode when VO_2_ is in the metallic state. Two conversion bands are identified for circular polarization within the frequency bands of 0.93–1.67 THz and 1.80–1.86 THz. The dual-mode polarization converter achieves axial ratios below 3 dB and a polarization conversion efficiency greater than 0.8. Surface current distributions reveal the polarization conversion mechanisms. Furthermore, we analyze the polarization ellipses of both reflected and transmitted waves at various frequencies across the operational bands. We anticipate that the proposed design, featuring high performance and dual functionality, will be applicable in THz communication systems and sensors.

## Introduction

Terahertz (THz) waves are found in the 0.1–10 THz spectral range, which is between the microwave and infrared bands. This spectral band is attractive for different applications such as 6G-wireless communications, imaging, THz-based gas, and biomedical sensors^1,2^. High-performance THz functional devices such as absorbers, filters, modulators, and polarization converters (PCs) are needed for these applications to manipulate THz waves^3–5^. Devices that control the polarization of THz waves are particularly crucial for a variety of applications, including sensors and communication systems^6,7^. The birefringence of uniaxial crystalline materials is generally required for conventional polarization optics, which results in phase retardations between the two orthogonally polarized components^8,9^. However, these natural crystals are restricted in the THz band because of their poor birefringence, narrow bandwidth, high loss, and enormous volume. Thus, to fully control the polarization state of the THz waves, more practical and adaptable methods are preferred. One of these methods is to use metamaterials-based PCs. Metamaterials can be defined as artificial composite materials engineered in the form of periodic metal/dielectric-based subwavelength structures. As a result, it possesses special electromagnetic attributes that are not present in natural materials. These unique features offer an alternative method of controlling the polarization of electromagnetic waves^5,10–18^. Scanning the technical literature, several theoretical and experimental studies have been introduced in designing metamaterials-based THz wave PCs in transmission, reflection modes, or both^19–28^. Each type of PC has its own set of advantages and disadvantages. The choice of the most appropriate type depends on the specific application requirements and design constraints. Although metamaterials have exhibited exceptional polarization conversion capabilities, research has primarily concentrated on either transmission-mode or reflection-mode operation. Dual-mode converters provide the most versatility, but research on metamaterials-based PCs operating in transmission and reflection modes is rare.

To convert a THz wave polarization mode from a TM to a TE, Xu et al. experimentally investigated a compound metasurface integrated with an H-shaped metallic metamaterial and a 45-degree-arranged subwavelength dielectric grating on the two sides of a silicon substrate^19^. Ako et al. proposed a free-standing, three-layer linear-to-linear (LTL) THz PC in transmission mode^20^. Split-ring and H-shaped resonators are situated between two layers of orthogonal wire gratings in the proposed device. A dielectric material was chosen to be a cyclic olefin copolymer. Over 0.2–1.0 THz, the measurement showed an ultra-wide bandwidth of 133% with a polarization conversion efficiency (PCE) > 80%. Based on the metasurface, Wang et al. suggested and verified experimentally a linear-to-circular (LTC) wideband PC in the THz band working in transmission mode^21^. A wire grid, a dielectric layer, and two pairs of symmetrical meandering lines made up the PC. In the frequency band of 0.4–1.21 THz, it possesses a relative bandwidth of almost 100% and axial ratio (AR) < 3 dB.

A reflecting LTC-PC was proposed by Jiang et al.^22^. The converter consists of a metal ground, a polyimide dielectric material, and an anisotropic double-split resonant square ring. It works with an AR ≤ 3dB and PCE ≥ 88% in the frequency band 0.60–1.41 THz. Moreover, the proposed design is capable of operating with PCE > 82% in the frequency band of 1.48–1.54 THz. Yin et al. proposed two J-shaped resonators to serve as chiral metamaterial structures to manipulate circular dichroism, asymmetric reflection of circularly polarized (CP) waves, linear-to-elliptical polarization conversion, and asymmetric transmission of linearly polarized (LP) waves inside the THz domain^23^.

To enhance miniaturization and system integration, researchers are exploring the integration of polarization manipulation in both reflection and transmission modes. The common idea is to use phase-transition materials such as vanadium dioxide (VO_2_)^24–26,29^ or photosensitive semiconductors such as silicon (Si) or germanium (Ge)^27^ or both of them^28^ for switching the polarization conversion between the transmission and the reflection modes. For instance, a bilayered metamaterial embedded with VO_2_ was proposed by Zou et al. as a double-use linear polarization converter^24^. One benefit of the suggested device is its ability to switch between transmission and reflection polarization conversion depending on the VO_2_ film’s phase transition in the THz range. In both situations, the polarization conversion ratios are greater than 90% in broad bands. Zhao et al. presented a dynamically switchable broadband dual-mode PC composed of a silicon dioxide (SiO_2_) dielectric spacer, a metal resonator, a double-layer graphene grating, and an alternating Au-VO_2_ grating^25^.

Tian et al. created a multifunctional optically controlled PC metasurface by adding photosensitive semiconductors Si and Ge to the resonator. Pump light at wavelengths of 1550 nm (for Ge excitation) and 800 nm (for Si excitation) is essential for transitioning between reflecting and transmissive modes^27^. The enhancement of the working efficiency of the proposed structure was controlled by the intensity of the pump light. In addition, a dual-mode metasurface PC employing Si and VO₂ was presented by Zhao et al.^28^. VO₂ was used in its insulating state to achieve LTL polarization conversion in transmission mode. In reflection mode, LTL and LTC polarization conversion were achieved by modulating the state of the Si by controlling the intensity of the pump light while VO₂ was insulating.

In this study, based on phase-transition material VO_2_, we present a design of LTC-PC in reflection and transmission modes. In the metallic state, the polarizer acts in reflection mode. This mode converts the LP incident wave (in the *x*- or *y*-direction) into a CP wave. Two conversion bands for right-hand circular-polarization (RH) or left-hand (LH) circular-polarization are obtained with an AR ≤ 3dB; in the frequency bands of 0.93–1.67 THz and 1.80–1.86 THz, corresponding to a relative bandwidth of 57% and 3.3%, respectively. In a dielectric state, the polarizer functions in transmission mode, converting the LP incident wave traveling along the *x*- or *y*-direction into a CP wave. With an AR ≤ 3dB, we obtain two conversion bands with relatively small bandwidths: from 1.26 to 1.47 THz and 1.83 to 1.85 THz. Moreover, a single conversion frequency is obtained at 1.7 THz. The proposed structure is suitable for applications including THz communication systems, sensors, and imaging systems.

The rest of this paper is organized as follows: The suggested dual-mode LTC-PC structure, materials, fabrication method, numerical simulations, and comprehensive mathematical concepts of the polarization conversion process are all covered in detail in Sect. 2. The obtained simulation results for both conversion modes are illustrated in Sect. 3. Section 4 discusses the physical mechanisms underlying the achieved results. A comparison between the proposed dual-mode LTC-PC with the recently reported dual-mode PCs is stated in Sect. 5. Finally, Sect. 6 concludes the major points.

## Materials, structure, and methodology

### Material models

The suggested LTC-PC is primarily composed of three different materials. The first one is the SiO_2,_ which acts as a dielectric material in the proposed design. In the present work, the SiO_2_ is modeled as a lossless dielectric material with a constant relative permittivity of 3.8 in the THz band under consideration (i.e., up to 2 THz)^30–32^. The second type of material used in the proposed design is gold (Au). Au is modeled as a lossy metal with a constant conductivity of $\:4.56\times\:{10}^{7}$ [S/m]. The last type of material is the VO_2,_ which is a transition material. By controlling the temperature of the VO_2_ layer, the mode of operation of the proposed structure can be tuned. At room temperature, VO_2_ acts as a dielectric, and the structure will perform the LTC conversion in the transmission mode. At temperatures nearly equal to or greater than 350 K, VO_2_ acts as a metal, and the device will perform the LTC conversion in reflection mode. The effective relative permittivity of VO_2_ is described by the following Drude model^10,33,34^:

1-a $$\:{\varepsilon\:}_{\text{V}\text{O}2}\left(\omega\:\right)={\varepsilon\:}_{{\infty\:}}-\frac{{\omega\:}_{p}^{2}\left(\sigma\:\right)}{{\omega\:}^{2}+i{\Gamma\:}{\upomega\:}}$$

where $\:{\varepsilon\:}_{\infty\:}$ is the dielectric constant at high frequency and equal to 12. $\:{\Gamma\:}$ denotes the scattering rate and is equal to $\:5.75\times\:{10}^{13}$ [rad/s]. $\:{\omega\:}_{p}\left(\sigma\:\right)$ is the plasma frequency as a function of VO_2_ conductivity, $\:\sigma\:$, which is given by:

1-b $$\:{\omega\:}_{p}\left(\sigma\:\right)=\sqrt{\frac{\sigma\:}{{\sigma\:}_{0}}{\omega\:}_{p}^{2}\left({\sigma\:}_{0}\right)}$$

where the constants $\:{\sigma\:}_{0}$ and $\:{\omega\:}_{p}\left({\sigma\:}_{0}\right)$ are equal to $\:3\times\:{10}^{5}$ [S/m] and $\:1.4\times\:{10}^{15}$ [rad/s], respectively. At $\:T\:=\:300\:\text{K}$ (i.e., VO_2_ acts as an insulator), $\:\sigma\:$ is assumed to be equal to 20 [S/m]. At $\:T\cong\:350\:\text{K}$ (i.e., VO_2_ acts as a metal), $\:\sigma\:$ is assumed to be equal to $\:2\times\:{10}^{5}$ S/m. Thus, the plasma frequency of the VO_2_ at the insulator case is $\:1.1431\times\:{10}^{13}$ [rad/s] while at the metal case is $\:1.1431\times\:{10}^{15}$ [rad/s].

### Structure description

**Fig. 1:**
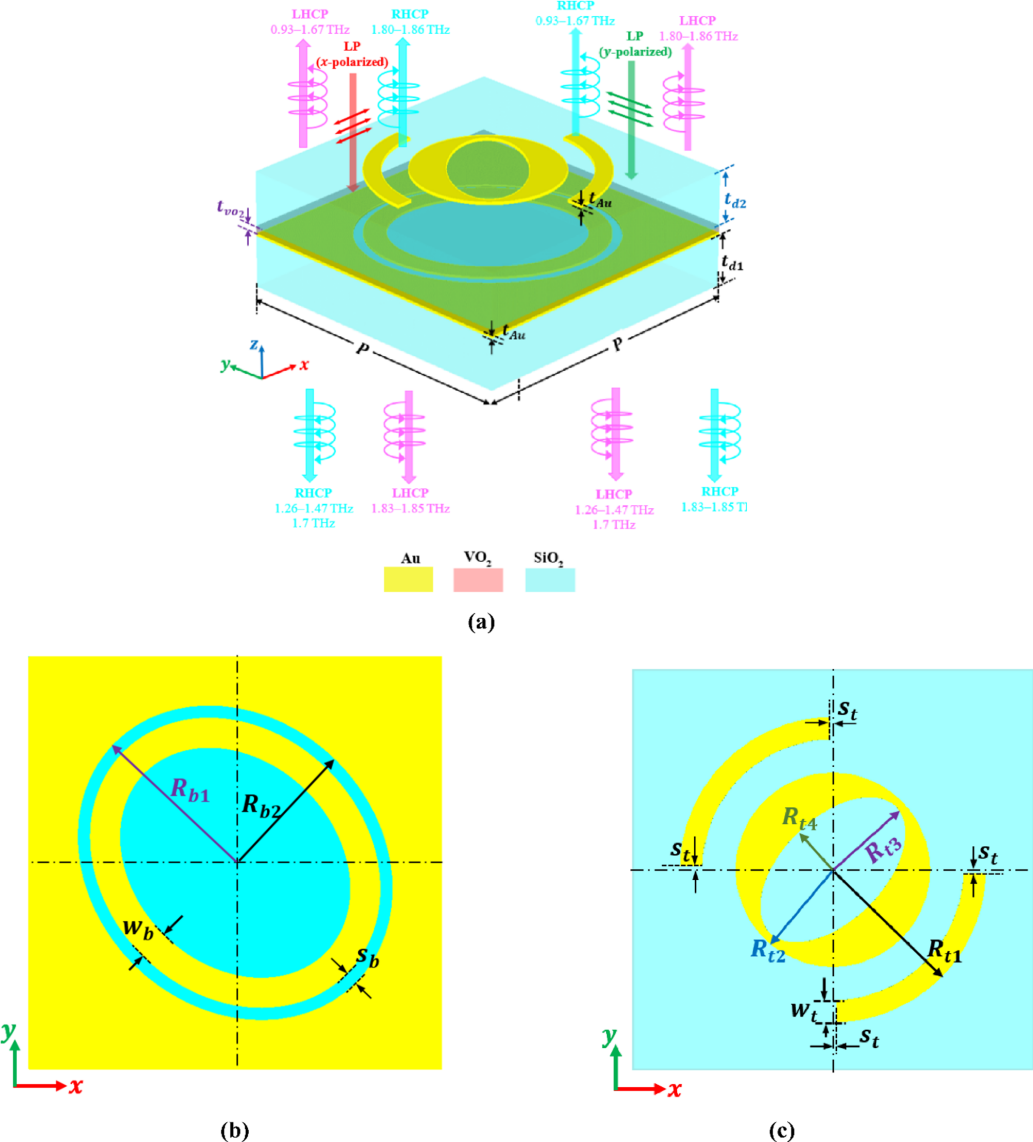
Proposed polarization conversion unit cell. (**a**) Schematic diagram, (**b**) Back Au pattern, (**c**) Top Au pattern.

**Fig. 2:**
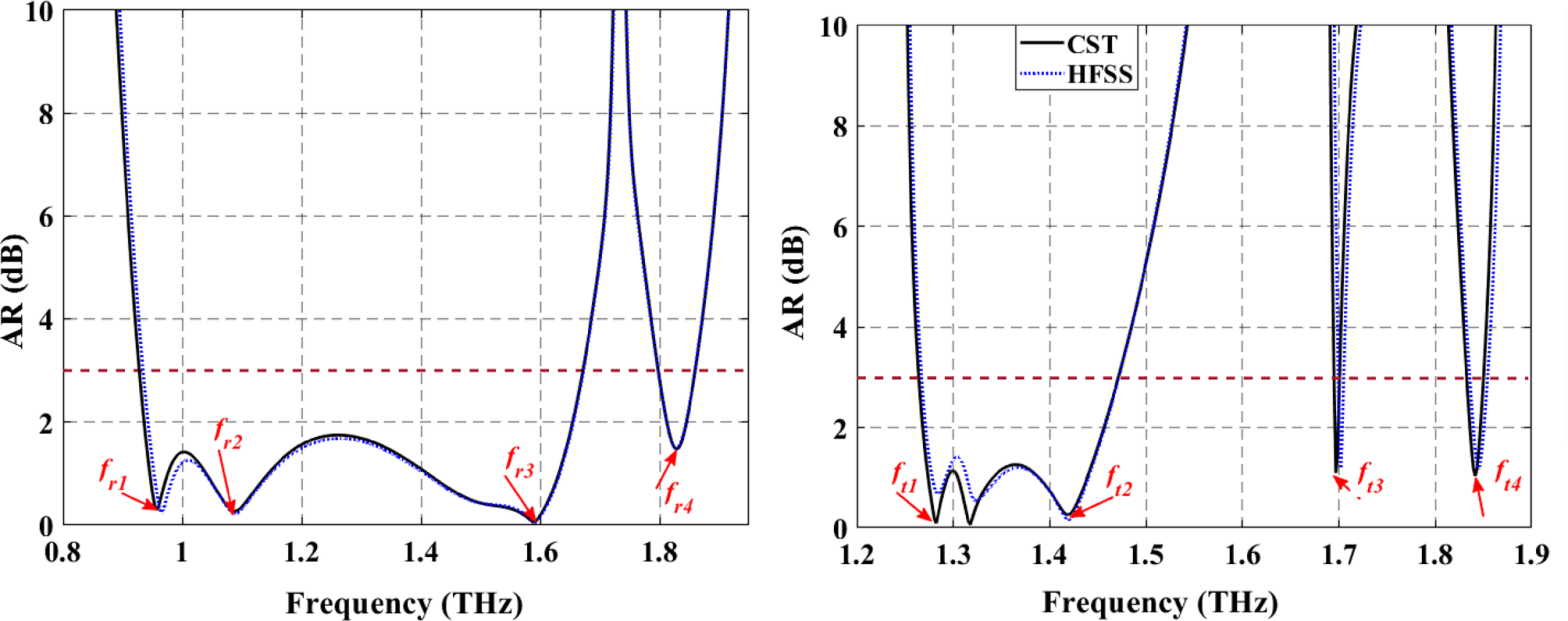
AR spectrum of the proposed LTC-PC unit cell by using CST and HFSS for (**a**) Reflection mode. (**b**) Transmission mode.

The proposed structure is periodic along the $\:x$ and $\:y\:$directions with a period equal to $\:P$. Figure 1a shows the schematic view of one unit cell of the proposed structure. This unit cell is composed of three layers, which can be described from bottom to top as follows: the first layer is composed of a SiO_2_ substrate layer with a thickness $\:{t}_{d1}$ and an Au pattern with a thickness $\:{t}_{Au}$. This Au pattern is called the back Au pattern, as illustrated in Fig. 1b. This pattern is composed of an Au layer perforated by an elliptical hole with a concentric elliptical ring of Au inside it. The major and minor radii of the elliptical hollow are $\:{R}_{b1}$ and $\:{R}_{b2}$, respectively. The thickness of the elliptical ring is $\:{w}_{b}$. The ring is away from the elliptical border by a distance $\:{s}_{b}$. The second layer is a VO_2_ layer with a thickness of $\:{t}_{{vo}_{2}}$, where the Au pattern from the first layer is engraved onto it. The third layer is composed of a SiO_2_ layer with a thickness of $\:{t}_{d2}$ and an Au pattern with a thickness $\:{t}_{Au}$. The Au pattern depicted in Fig. 1c is referred to as the top Au pattern. This pattern consists of a circular disk with an elliptical hollow inside. The radius of the circular disk is $\:{R}_{t2}$. The major and minor radii of the elliptical hollow are $\:{R}_{t3}$ and $\:{R}_{t4}$, respectively. This circular disk is surrounded by double symmetrical semi-quarter rings with an outer radius of $\:{R}_{t1}$ and width $\:{w}_{t}$. Each semi-quarter ring is $\:{S}_{t}$ away from the $\:x$- or $\:y$-axis. It should be noted that the shape, materials, initial design decisions, and parameter values are selected using a combination of physical knowledge and symmetry considerations. The design parameters are then optimized utilizing a trial-and-error technique and a parametric sweep to improve polarization conversion efficiency across the intended frequency range. The obtained results in this work are based on the optimum design parameters given in Table 1. These parameters are optimized to maintain an AR of less than 3 dB in the reflection mode and transmission mode, as will be illustrated later in Fig. 2.

By altering the conductivity of the VO_2_ material through thermal treatment, the suggested structure’s switchable capabilities from reflection mode to transmission mode can be accomplished^35^. The insulator state of VO_2_ is maintained at room temperature. External heating^36^, electrical bias (i.e., through the current-induced Joule heating effect)^34,37–40^, high-intensity THz field^41^, or optical excitation^42^ can all be used to control the phase transition property of VO_2_. Classical external heating can effectively control the phase transition of the VO_2_ layer in the proposed LTC-PC structure. Furthermore, the continuous design of the back Au pattern under the VO_2_ layer (see Fig. 1b) allows for control of the VO_2_ phase transition in the proposed LTC-PC structure using an external electrical bias^10^. The bias voltage generates a uniform current through the metamaterial, raising the temperature of the VO_2_ layer due to the current-induced Joule heating effect in the metal structure. Relying on external heat to switch the phase of VO_2_ is a limitation. The necessity for an external heater makes VO_2_-based THz switchable devices costly and bulky. In contrast, using electrical control for the phase transition of VO_2_ is less constrained and more practical for real-world applications. The suggested structure can thus function in transmission mode as a result of the incoming THz wave interacting with a top Au pattern-SiO_2_ layer and propagating via the bottom Au pattern-SiO_2_ layer. When the temperature goes above the phase-change temperature, VO_2_ becomes fully metallic and highly conductive, which means the bottom Au pattern-VO_2_ becomes a solid metallic layer that can block transmission. This configuration can therefore function in reflection mode.

**Table 1:** Optimum design parameters of the proposed THz LTC-PC (Units in $\:\mu\:m$).

$\:\varvec{P}$	126	$\:{\varvec{R}}_{\varvec{t}1}$	48	$\:{\varvec{R}}_{\varvec{b}1}$	52
$\:{\varvec{t}}_{\varvec{d}1}$	22	$\:{\varvec{R}}_{\varvec{t}2}$	30.5	$\:{\varvec{R}}_{\varvec{b}2}$	44
$\:{\varvec{t}}_{\varvec{d}2}$	24	$\:{\varvec{R}}_{\varvec{t}3}$	28	$\:{\varvec{s}}_{\varvec{b}}$	4
$\:{\varvec{t}}_{\varvec{v}\varvec{o}2}$	2	$\:{\varvec{R}}_{\varvec{t}4}$	16	$\:{\varvec{w}}_{\varvec{b}}$	9
$\:{\varvec{t}}_{\varvec{A}\varvec{u}}$	0.2	$\:{\varvec{s}}_{\varvec{t}}$	1		

**Fig. 3:**
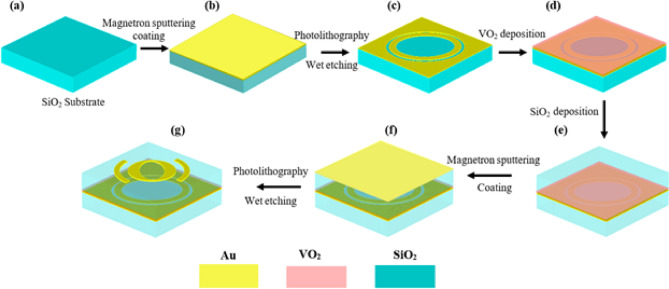
Fabrication steps of the proposed dual-mode LTC-PC.

Drawing on prior research, we propose the subsequent fabrication technique for the suggested LTC-PC^43–45^. Initially, a typical SiO_2_ substrate is prepared, as illustrated in Fig. 3a. A layer of Au is subsequently placed on the SiO_2_ layer via magnetron sputtering, as illustrated in Fig. 3b. The bottom Au pattern is defined using traditional photolithography and wet etching, as depicted in Fig. 3c. Subsequently, the VO_2_ layer may be deposited onto the Au pattern using techniques such as reactive magnetron sputtering^46–48^, sol–gel with dip-coating^49–51^, or spin-coating^43^, followed by annealing. The coating procedure is reiterated to achieve the desired VO_2_ film thickness, as seen in Fig. 3d. Thereafter, an additional layer of SiO_2_ is applied to the VO_2_ layer by sol–gel techniques using either spin-coating^52,53^ or dip-coating^54,55^, followed by annealing. Multiple coats may be deposited to achieve the desired thickness, as illustrated in Fig. 3e. A layer of Au is subsequently placed on the SiO_2_ layer via magnetron sputtering, as depicted in Fig. 3f. The final Au pattern is created using traditional photolithography and wet etching, as illustrated in Fig. 3g.

### Methodology and theoretical principles

The following section provides the numerical methods employed to obtain our results in detail. The electromagnetic simulations of the proposed structure are performed using the finite-element method with a frequency-domain solver from the commercial software CST Studio. Since the structure is periodic, we simulate only one unit cell, setting the boundary conditions to represent a unit cell in both the *x* and *y* directions. In the positive and negative *z*-directions, the boundary conditions are configured to be open (add space). We enable adaptive mesh refinement to ensure high accuracy for the results obtained. Furthermore, during the simulation process, the plasma frequency and conductivity of VO_2_ are set following Eq. 1-a and 1-b. It is preferred to use a direct solver in the simulations because the Au is modeled as a lossy metal.

For the verification of the obtained simulation results, we carried out simulations on the same unit cell by using commercial software HFSS. To simulate the periodicity of the structure in HFSS, the structure is bounded by the two master-slave boundaries parallel to $\:xz$- and $\:yz$-planes with an open space of 88$\:\:\mu\:m$ added between the structure and the illumination ports. This structure is illuminated by Floquet ports along *x*- or -directions with a normal incident wave along the $\:\pm\:z$ direction at both ends. To accurately model the VO_2_ in HFSS and simulate its electromagnetic behavior, its permittivity should be defined, which is frequency dependent and complex-valued as calculated by Eq. 1. The spectral response of AR of the proposed LTC-PC structure under incidence of $\:x$-polarized wave using CST and HFSS is shown in Fig. 2. This figure indicates the good agreement between the simulated AR by using the two simulators. The AR of the reflection mode shows two circular polarization bands as depicted in Fig. 2a. Four points of nearly-perfect circular polarization of the reflected waves with AR $\:\ll\:$ 3 dB are observed at frequencies of $\:{f}_{r1}$= 0.96 THz,$\:\:{f}_{r2}$= 1.10 THz, $\:{f}_{r3}$= 1.59 THz and $\:{f}_{r4}$= 1.82 THz. In the transmission mode, two bands and a single frequency for LTC polarization conversion are observed as in Fig. 2b. In this mode, an LTC polarization conversion in the frequency bands of 1.26 to 1.47 THz and 1.83 to 1.85 THz is obtained. Furthermore, this mode yields an LTC polarization conversion at a frequency of 1.7 THz. Four points of nearly-perfect circular polarization of transmitted waves are observed at frequencies of $\:{f}_{t1}$= 1.28 THz,$\:\:{f}_{t2}$= 1.42 THz, $\:{f}_{t3}$= 1.70 THz and $\:{f}_{t4}$= 1.84 THz. To calculate the value of the AR, some mathematical concepts of the polarization conversion process should be studied first.

An x- or y-polarized wave is presumed to be incident downward on the proposed structure from + *z* to -*z* to gain a more comprehensive understanding of the polarization conversion process for the LTC-PC, as illustrated in Fig. 1a. Based on polarization conversion theory, the reflected electric field can be related to the incident electric field by the LTL reflective matrix $\:{\text{R}}_{LTL}$ as^56–58^:

2 $$\:\left[\begin{array}{c}{E}_{x}^{r}\\\:{E}_{y}^{r}\end{array}\right]={\text{R}}_{LTL}\left[\begin{array}{c}{E}_{x}^{i}\\\:{E}_{y}^{i}\end{array}\right]=\left[\begin{array}{cc}\begin{array}{c}{R}_{xx}\\\:{R}_{yx}\end{array}&\:\begin{array}{c}{R}_{xy}\\\:{R}_{yy}\end{array}\end{array}\right]\left[\begin{array}{c}{E}_{x}^{i}\\\:{E}_{y}^{i}\end{array}\right]$$

where $\:\left({E}_{x}^{i},\:{E}_{y}^{i}\right)$ and $\:\left({E}_{x}^{r},\:{E}_{y}^{r}\right)$ are the complex amplitudes of the incident and reflected wave components in the $\:x$ and $\:y$ directions, respectively. $\:{R}_{xx}$ and $\:{R}_{yy}$ are the co-polarized reflection coefficients which can be represented as; $\:{R}_{xx}={r}_{xx}{e}^{{\phi\:}_{r\_xx}}$ and $\:{R}_{yy}={r}_{yy}{e}^{{\phi\:}_{r\_yy}}$. Here, $\:{r}_{xx}=\left|{E}_{x}^{r}\right|/\left|{E}_{x}^{i}\right|$ and $\:{r}_{yy}=\left|{E}_{y}^{r}\right|/\left|{E}_{y}^{i}\right|$ are the magnitude of the co-polarized reflection coefficients. Also, $\:{R}_{yx}$ and $\:{R}_{xy}$ are the cross-polarized reflection coefficients which can be represented as; $\:{R}_{yx}={r}_{yx}{e}^{{\phi\:}_{r\_yx}}$ and $\:{R}_{xy}={r}_{xy}{e}^{{\phi\:}_{r\_xy}}$. $\:{r}_{yx}=\left|{E}_{y}^{r}\right|/\left|{E}_{x}^{i}\right|$ and $\:{r}_{xy}=\left|{E}_{x}^{r}\right|/\left|{E}_{y}^{i}\right|$ are the magnitudes of the cross-polarized reflection coefficients. To achieve circular polarization from the co- and cross-polarized reflected components, two conditions should be satisfied. The first one, the magnitudes of the co-polarized reflection coefficients, are comparable to the magnitudes of the cross-polarized reflection coefficients. The second condition is related to the phase difference between the co- and cross-polarized reflection coefficients $\:\varDelta\:{{\upvarphi\:}}_{r}$, which should be around $\:\pm\:90^\circ\:$ resulting in either LH or RH circular polarization. The phase difference is mainly defined as the phase by which the $\:y$-component leads the -component^58^. Thus, under $\:x$-polarized wave, the phase difference can be calculated as $\:\varDelta\:{{\upvarphi\:}}_{rx}={\phi\:}_{yx}-{\phi\:}_{xx}$. Under $\:y$-polarized wave, the phase difference can be calculated as $\:\varDelta\:{{\upvarphi\:}}_{ry}={\phi\:}_{yy}-{\phi\:}_{xy}$.

Moreover, the CP reflected electric fields can be related to the incident electric field by the LTC reflective matrix $\:{\text{R}}_{LTC}$ as:

3 $$\:\left[\begin{array}{c}{E}_{L}^{r}\\\:{E}_{R}^{r}\end{array}\right]={\text{R}}_{LTC}\left[\begin{array}{c}{E}_{x}^{i}\\\:{E}_{y}^{i}\end{array}\right]$$

4 $$\:{\text{R}}_{LC}=\frac{1}{\sqrt{2}}\left[\begin{array}{cc}\begin{array}{c}{R}_{LH-x}\\\:{R}_{RH-x}\end{array}&\:\begin{array}{c}{R}_{LH-y}\\\:{R}_{RH-y}\end{array}\end{array}\right]=\frac{1}{\sqrt{2}}\left[\begin{array}{cc}\begin{array}{c}{R}_{xx}-i{R}_{yx}\\\:{{R}_{xx}+iR}_{yx}\end{array}&\:\begin{array}{c}{{R}_{xy}-iR}_{yy}\\\:{R}_{xy}+i{R}_{yy}\end{array}\end{array}\right]$$

where, $\:{R}_{LH-x}$ and $\:{R}_{RH-x}$ are LH and RH reflection coefficients of the reflected CP wave under the incidence of $\:x$-polarized wave. Also, $\:{R}_{LH-y}$ and $\:{R}_{RH-y}$ are LH and RH reflection coefficients of the CP wave under the incidence of $\:y$-polarized wave. To numerically evaluate the circular polarization conversion performance of the reflected wave, an important evaluation parameter named PCE is employed to assess the quality of the conversion for both LH and RH, assuming $\:x$-polarized incident waves, as:

5-a $$\:{\text{P}\text{C}\text{E}}_{LH-x}=\frac{{\left|{R}_{LH-x}\right|}^{2}}{{\left|{R}_{RH-x}\right|}^{2}+{\left|{R}_{LH-x}\right|}^{2}}$$

5-b $$\:{\text{P}\text{C}\text{E}}_{RH-x}=\frac{{\left|{R}_{RH-x}\right|}^{2}}{{\left|{R}_{RH-x}\right|}^{2}+{\left|{R}_{LH-x}\right|}^{2}}$$

The objective of a nearly-perfect circular polarizer is to ensure that one reflected CP wave is significantly larger or smaller than the other, as evidenced by the following: $\:{R}_{LH-x}\gg\:{R}_{RH-x}$ or $\:{R}_{LH-x}\ll\:{R}_{RH-x}$. This implies that $\:{\text{P}\text{C}\text{E}}_{LH-x}\approx\:1$ or $\:{\text{P}\text{C}\text{E}}_{RH-x}\approx\:1$ for LH or RH polarization conversion, respectively.

In addition, an important parameter to evaluate the circular polarization is the AR. It can be determined using two distinct approaches. One method relies on the magnitudes of the orthogonal linear components of the reflected wave, along with their phase difference. This approach can be calculated, assuming linearly $\:x$-polarized incident waves, as [p.21 in 61]:

6 $$\:{AR}_{rx}\left(\text{d}\text{B}\right)=20{\text{l}\text{o}\text{g}}_{10}\left(\sqrt{\frac{{{r}_{xx}}^{2}+{{r}_{yx}}^{2}+\sqrt{a}}{{{r}_{xx}}^{2}+{{r}_{yx}}^{2}-\sqrt{a}}}\right)$$

where;

7 $$\:a={{r}_{xx}}^{4}+{{r}_{yx}}^{4}+2{{r}_{xx}}^{2}{{r}_{yx}}^{2}\text{cos}\left(2\varDelta\:{{\upvarphi\:}}_{rx}\right)$$

Alternatively, the AR can be derived from the magnitudes of the RH and LH components of the reflected waves $\:{R}_{RH-x}$ and $\:{R}_{LH-x}$. This method directly utilizes the decomposed circular polarization components, providing a complementary perspective on the polarization characteristics. The AR can be calculated using the following expression [p.37 in^59^]:

8 $$\:{AR}_{rx}\left(\text{d}\text{B}\right)=20{\text{l}\text{o}\text{g}}_{10}\left(\frac{{R}_{RH-x}+{R}_{LH-x}}{{R}_{RH-x}-{R}_{LH-x}}\right)$$

To provide a complete description of the polarization state of the reflected CP wave, three key parameters are used. These parameters are the ellipticity angle ($\:{\beta\:}_{rx}$), ellipticity ($\:{\chi\:}_{rx}$) and tilted angle ($\:{\tau\:}_{rx}$). Together, these parameters define the shape, orientation, and phase properties of the polarization ellipse, which describe the polarization nature of the reflected wave [p.43 in^59^]. These parameters are calculated, assuming linearly $\:x$-polarized incident waves, as:

9 $$\:{\beta\:}_{rx}=0.5{\text{sin}}^{-1}\left(\text{sin}2{\gamma\:}_{rx}\text{sin}\varDelta\:{{\upvarphi\:}}_{rx}\right)$$

10 $$\:{\chi\:}_{rx}=\text{sin}\left(2{\beta\:}_{x}\right)$$

11 $$\:{\tau\:}_{rx}=0.5\text{t}{\text{an}}^{-1}\left(\frac{\text{sin}2{\gamma\:}_{rx}\text{cos}\varDelta\:{{\upvarphi\:}}_{rx}}{\text{cos}2{\gamma\:}_{rx}}\right)$$

where the angle $\:{\gamma\:}_{x}$ relates the magnitudes of the co- and cross-polarized reflected components and is calculated as $\:{\gamma\:}_{rx}\:={\text{tan}}^{-1}\left(\frac{{r}_{yx}}{{r}_{xx}}\right)$ [p. 31 in^59^]. For the perfect LH (or RH) CP wave, the ellipticity angle is 45° (or $\:-45^\circ\:$) corresponding to ellipticity of + 1 (or $\:-1$), respectively. Equations (5)–(11) apply to linearly $\:x$-polarized incident waves. Equations for the linearly $\:y$-polarized wave can be defined similarly.

**Fig. 4:**
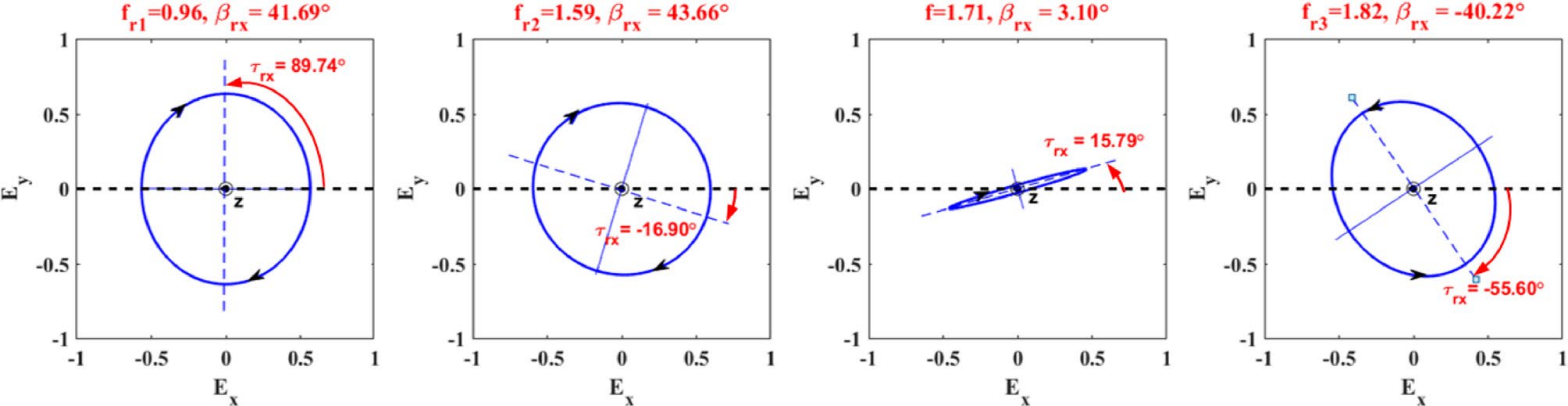
Trajectory of the reflected wave at different frequencies. The observer is facing the field’s source, as indicated by the filled circle at the origin.

dNow, several polarization states for $\:x$-polarized incidence wave are plotted at different frequencies as provided in Fig. 4. The polarization ellipse signifies the rotation sense, ellipticity angle, and tilt angle of the reflected polarized wave. The polarization ellipses have been generated using the magnitudes and phases of reflection coefficients of co- and cross-polarized waves $\:{r}_{xx}$ and $\:{r}_{yx}$, respectively. From this figure, at frequencies of 0.96 and 1.59 THz, the polarization ellipses are approximately viewed as circles with an ellipticity around 1 and ellipticity angles around 45° meaning that the reflected waves are LH with AR$\:\approx\:0\:\text{d}\text{B}$. Moreover, it can be observed that the incident and reflected wave have the same linear polarization along $\:x$-direction at an arbitrarily chosen frequency of 1.71 THz with an ellipticity of around 0, an ellipticity angle of 3.10° and a tilted angle of 15.79° with respect to the $\:x$-axis. At a frequency of 1.82 THz, the reflected wave becomes RH, and its major axis rotates with respect to the $\:x$-axis with a tilted angle of $\:-$55.60°.

In the transmission mode of the proposed structure, the LTC-PC can be used for linearly $\:x$- or $\:y$-polarized incident waves. Correspondingly, in the same way, as given by Eq. 2, the transmitted electric field can be related to the incident electric field by the LTL transmissive matrix $\:{\text{T}}_{LTL}$ as:

12 $$\:\left[\begin{array}{c}{E}_{x}^{t}\\\:{E}_{y}^{t}\end{array}\right]={\text{T}}_{LTL}\left[\begin{array}{c}{E}_{x}^{i}\\\:{E}_{y}^{i}\end{array}\right]=\left[\begin{array}{cc}\begin{array}{c}{T}_{xx}\\\:{T}_{yx}\end{array}&\:\begin{array}{c}{T}_{xy}\\\:{T}_{yy}\end{array}\end{array}\right]\left[\begin{array}{c}{E}_{x}^{i}\\\:{E}_{y}^{i}\end{array}\right]$$

where $\:\left({T}_{xx},\:{T}_{yy}\right)$ and $\:\left({T}_{yx},\:{T}_{xy}\right)$ are the co-polarized and cross-polarized transmission coefficients under $\:x$- and $\:y$-polarized incident wave, respectively. Where, $\:{T}_{xx}={t}_{xx}{e}^{{\phi\:}_{t\_xx}}$ and $\:{T}_{yx}={t}_{yx}{e}^{{\phi\:}_{t\_yx}}$ where $\:{t}_{xx}=\left|{E}_{x}^{t}\right|/\left|{E}_{x}^{i}\right|$ and $\:{t}_{yx}=\left|{E}_{y}^{t}\right|/\left|{E}_{x}^{i}\right|$ are the magnitude of the co- and cross-polarized transmission coefficients under $\:x$-polarized incident wave. $\:{T}_{yy}={t}_{yy}{e}^{{\phi\:}_{t\_yy}}$ and $\:{T}_{xy}={t}_{xy}{e}^{{\phi\:}_{t\_xy}}$ where $\:{t}_{yy}=\left|{E}_{y}^{t}\right|/\left|{E}_{y}^{i}\right|$ and $\:{t}_{xy}=\left|{E}_{x}^{t}\right|/\left|{E}_{y}^{i}\right|$ are the magnitude of the co- and cross-polarized transmission coefficients under $\:y$-polarized wave. The phase difference between the two transmission coefficients can be calculated, under the incidence of the *x*-polarized wave, as $\:{\varDelta\:{{\upvarphi\:}}_{tx}=\phi\:}_{t\_yx}-{\phi\:}_{t\_xx}$ or $\:{\varDelta\:{{\upvarphi\:}}_{ty}=\phi\:}_{t\_yy}-{\phi\:}_{t\_xy}$. The CP transmitted electric field can be related to the incident electric field by the LTC transmissive matrix $\:{\text{T}}_{LTC}$ as:

13 $$\:\left[\begin{array}{c}{E}_{L}^{t}\\\:{E}_{R}^{t}\end{array}\right]={\text{T}}_{LTC}\left[\begin{array}{c}{E}_{x}^{i}\\\:{E}_{y}^{i}\end{array}\right]$$

14 $$\:{\text{T}}_{LTC}=\frac{1}{\sqrt{2}}\left[\begin{array}{cc}\begin{array}{c}{T}_{LH-x}\\\:{T}_{RH-x}\end{array}&\:\begin{array}{c}{T}_{LH-y}\\\:{T}_{RH-y}\end{array}\end{array}\right]=\frac{1}{\sqrt{2}}\left[\begin{array}{cc}\begin{array}{c}{T}_{xx}-i{T}_{yx}\\\:{{T}_{xx}+iT}_{yx}\end{array}&\:\begin{array}{c}{{T}_{xy}-iT}_{yy}\\\:{T}_{xy}+i{T}_{yy}\end{array}\end{array}\right]$$

where, $\:{T}_{LH-x}$,$\:{T}_{RH-x}$ are LH and RH transmission coefficients of the transmitted CP wave under the incidence of an *x*-polarized wave. Again, assuming linearly $\:x$-polarized incident waves, to quantify the circular polarization conversion performance of the transmitted wave, PCE is a key metric used to evaluate the quality of the conversion for both LH and RH circular polarizations as:

15-a $$\:{\text{P}\text{C}\text{E}}_{LH-x}=\frac{{\left|{T}_{LH-x}\right|}^{2}}{{\left|{T}_{RH-x}\right|}^{2}+{\left|{T}_{LH-x}\right|}^{2}}$$

15-b $$\:{\text{P}\text{C}\text{E}}_{RH-x}=\frac{{\left|{T}_{RH-x}\right|}^{2}}{{\left|{T}_{RH-x}\right|}^{2}+{\left|{T}_{LH-x}\right|}^{2}}$$

From the magnitudes of the transmitted CP components $\:{T}_{LH-x}$ and $\:{T}_{RH-x}$, it is easy to compute the $\:\text{A}\text{R}$ as:

16 $$\:{AR}_{tx}\left(\text{d}\text{B}\right)=20{\text{l}\text{o}\text{g}}_{10}\left(\frac{{T}_{RH-x}+{T}_{LH-x}}{{T}_{RH-x}-{T}_{LH-x}}\right)$$

To provide a complete description of the polarization state of the transmitted CP wave, we define the ellipticity ($\:{\chi\:}_{tx}$), ellipticity angle ($\:{\beta\:}_{tx}$) and tilted angle ($\:{\tau\:}_{tx}$), assuming linearly $\:x$-polarized incident waves, as follows:

17 $$\:{\beta\:}_{tx}=0.5{\text{sin}}^{-1}\left(\text{sin}2{\gamma\:}_{tx}\text{sin}\varDelta\:{{\upvarphi\:}}_{tx}\right)$$

18 $$\:{\chi\:}_{tx}=\text{sin}\left(2{\beta\:}_{tx}\right)$$

19 $$\:{\tau\:}_{tx}=0.5\text{t}{\text{an}}^{-1}\left(\frac{\text{sin}2{\gamma\:}_{tx}\text{cos}\varDelta\:{{\upvarphi\:}}_{tx}}{\text{cos}2{\gamma\:}_{tx}}\right)$$

where the angle $\:{\gamma\:}_{tx}$ relates the magnitudes of the co- and cross-polarized transmitted components given as $\:{\gamma\:}_{tx}\:={\text{tan}}^{-1}\left(\frac{{t}_{yx}}{{t}_{xx}}\right)$.

**Fig. 5:**
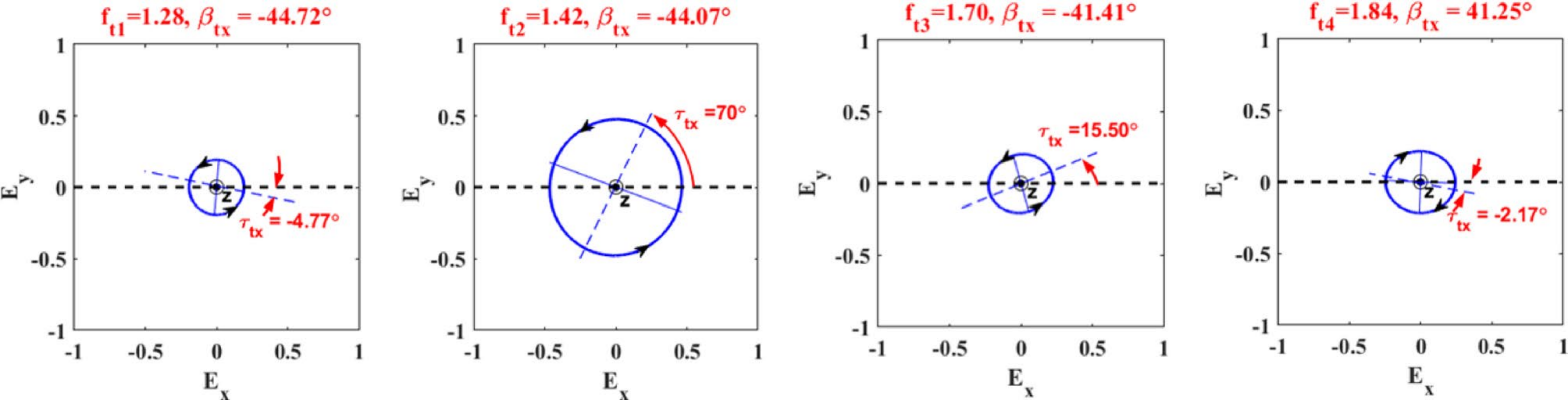
Trajectory of the transmitted wave at different frequencies. The observer is facing the field’s source, as indicated by the filled circle at the origin.

Figure 5 illustrates several polarization states corresponding to different frequencies for the transmitted CP wave under a linearly *x*-incident wave. We select four points of nearly perfect circular polarization for transmitted waves. The polarization ellipses effectively illustrate the LTC polarization conversion process. The gradual change in ellipticity, ellipticity angle, and rotation direction all point to a controlled manipulation of the polarization state of the incident wave.

## Results and discussion

### Reflection mode with VO_2_ layer in a metallic state

**Fig. 6:**
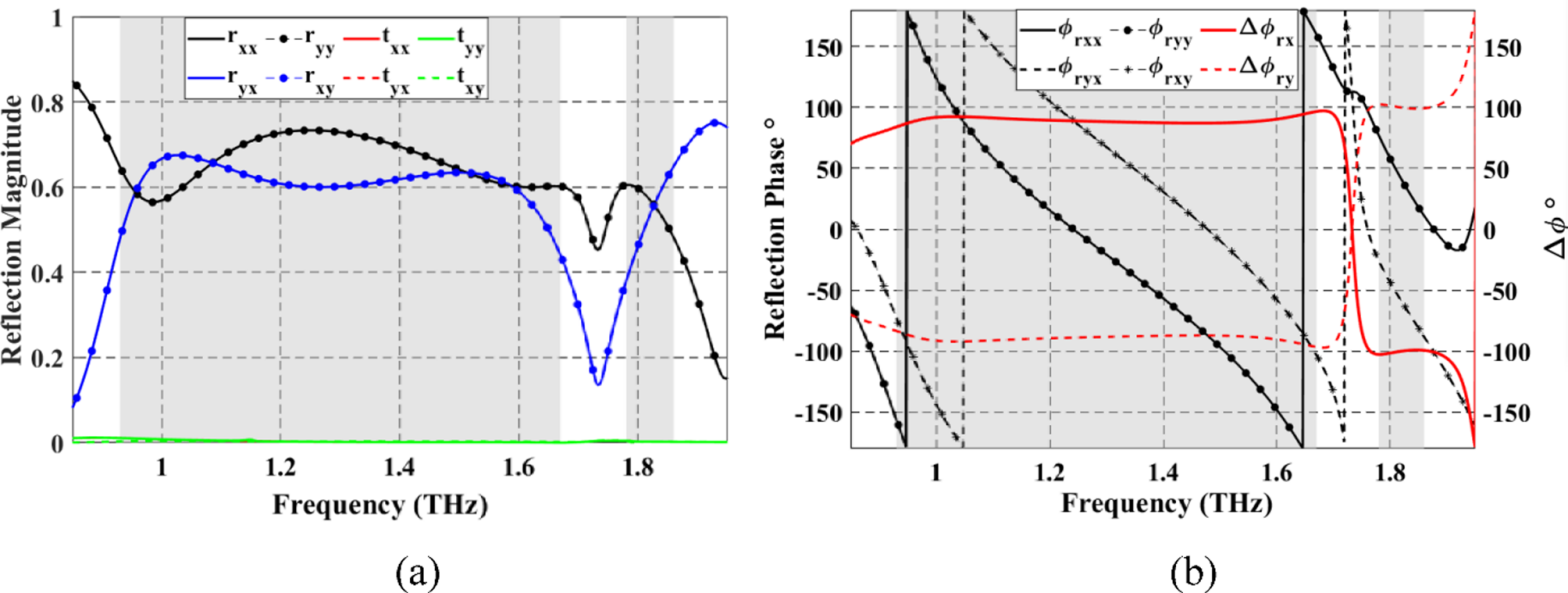
Spectrum of co- and cross-polarized reflection coefficients under $\:x$-polarized and $\:y$-polarized incident waves. (**a**) Reflection magnitude. (**b**) Reflection phase and phase difference. The CP-reflected waves’ frequency bands are shown by the gray regions.

Reflections from the proposed LTC-PC are simulated under both $\:x$- and $\:y$-polarized normal incident waves. When the VO_2_ layer is in the metallic state, it acts as a reflective plate that effectively blocks the transmission of electromagnetic waves. The results of the simulated co- and cross-polarized reflection and transmission coefficients are shown in Fig. 6a. In this figure, four resonance frequencies of co- and cross-polarization can be noted at $\:{f}_{r1}$= 0.96 THz,$\:\:{f}_{r2}$= 1.10 THz, $\:{f}_{r3}$= 1.59 THz and $\:{f}_{r4}$= 1.82 THz, where the co-reflection coefficients $\:{r}_{xx}=\:{r}_{yy}$ are the same as the cross-reflection coefficients $\:{r}_{yx}=\:{r}_{xy}$. These resonances indicate nearly-perfect circular polarization conversion of the reflected waves with AR $\:\ll\:$ 3 dB. Overall, $\:{r}_{xx},\:{r}_{yy}$ and $\:{r}_{yx},\:{r}_{xy}$ are both similar and carry more than 50% of incident power in the frequency range from 0.93 to 1.67 THz and from 1.80 to 1.86 THz with corresponding relative bandwidths of 57% and 3.3%, respectively. The similarity between the magnitudes of co- and cross-reflection coefficients is one of the two conditions of the circular polarization conversion. The second condition states that the corresponding phase difference must be around $\:\pm\:$90° as can be observed from Fig. 6b. Thus, under -normal incidence, the reflected wave has $\:\varDelta\:{{\upvarphi\:}}_{rx}$ of approximately 90° and $\:-$90° corresponding to an LH or RH wave in the two observation bands. Under *y*-normal incidence, the reflected wave is converted into RH and LH in the observation bands, as, $\:\:\varDelta\:{{\upvarphi\:}}_{ry}=-\varDelta\:{{\upvarphi\:}}_{rx}$.

**Fig. 7:**
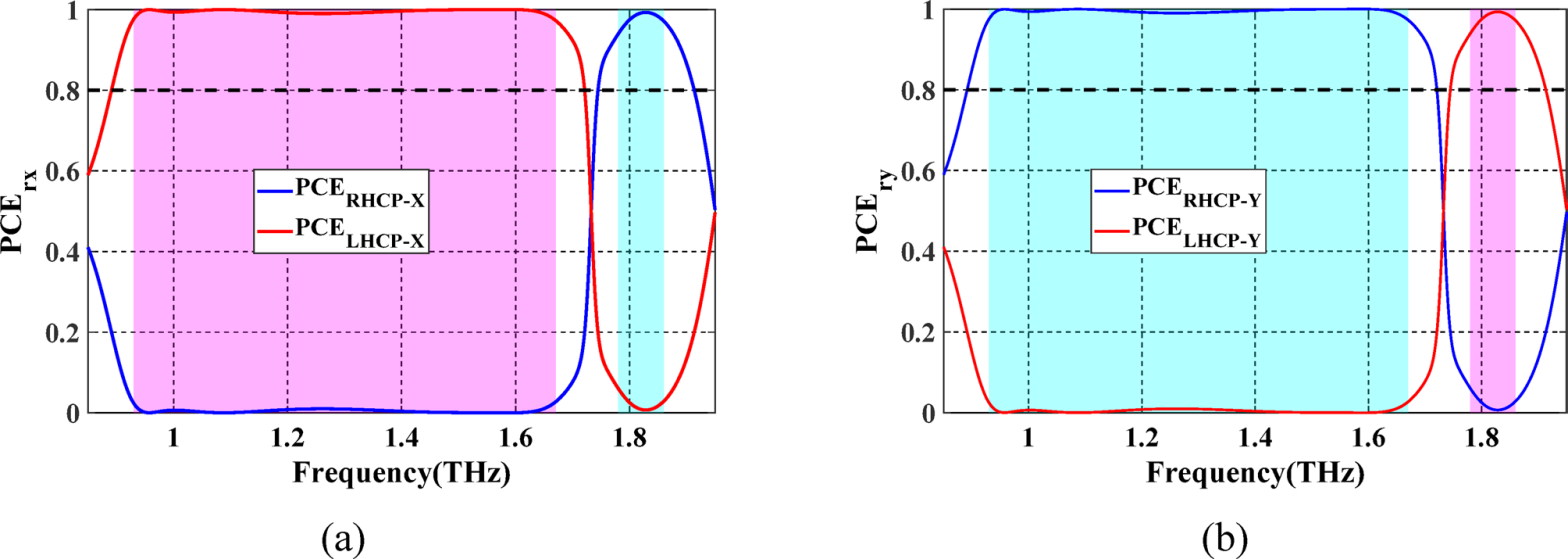
Spectrum of PCE of the proposed LTC-PC under (**a**) $\:x$-polarized incident wave and. (**b**) $\:y$-polarized incident wave. The frequency bands of the LH and RH reflected waves are shown by the pink and blue regions, respectively.

To decide the sense of the polarization conversion, the $\:{\text{P}\text{C}\text{E}}_{x}$ and $\:{\text{P}\text{C}\text{E}}_{y}$ for both LH and RH, ellipticity angles ($\:{\beta\:}_{rx}$ and $\:{\beta\:}_{ry}$) and ellipticity ($\:{\chi\:}_{rx}$ and $\:{\chi\:}_{ry}$) of the reflected waves are calculated according to Eqs. (9)–(11) and Eqs. (17)–(19), for $\:x$- and $\:y$-polarized waves, respectively. Figure 7a illustrates that the PCE values in the range of 0.93–1.67 THz and 1.80–1.86 THz are all greater than 80%. This result confirms the successful implementation of linear to LH and linear to RH circular polarization conversion, respectively, under $\:x$-polarized wave. Simultaneously, the PCE values in the same bands are greater than 80%, demonstrating the achievement of conversion from linear to RH and from linear to LH circular polarization, under $\:y$-polarized incident wave as indicated in Fig. 7b.

**Fig. 8:**
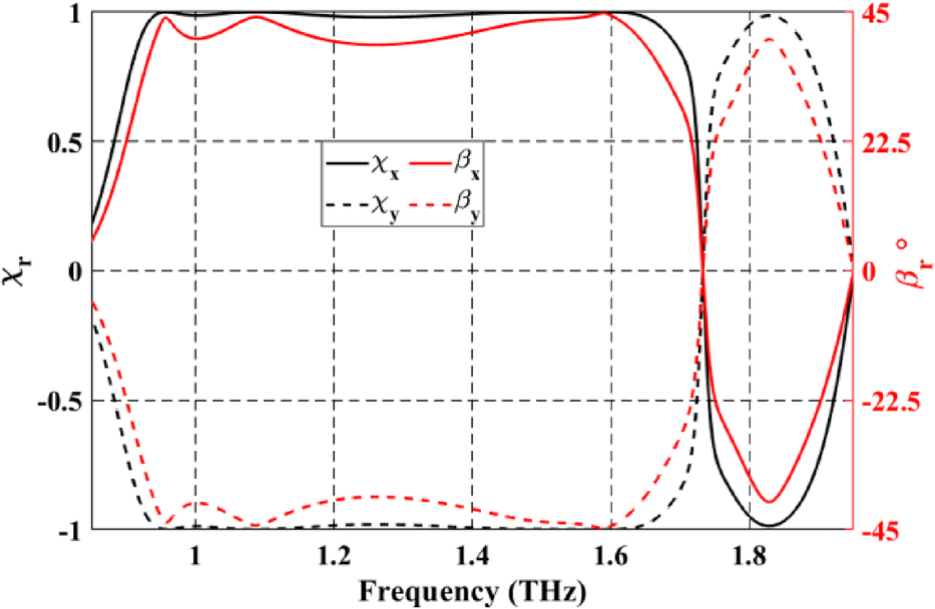
Spectrum of the ellipticity and elliptical angle under $\:x$-polarized and $\:y$-polarized incident waves.

On the other hand, as shown in Fig. 8, the values of both $\:{\chi\:}_{rx}$ and $\:{\beta\:}_{rx}$ are around (+ 1 and − 1) and around 45° and − 45° at the above-mentioned frequency bands, revealing that the reflected waves are LH and RH circular polarized under the incidence of the normal $\:x$-polarized wave. In addition, under the incidence of a normal $\:y$-polarized wave, the opposite is true, as clearly illustrated by the dashed red and black lines in Fig. 8.

**Fig. 9:**
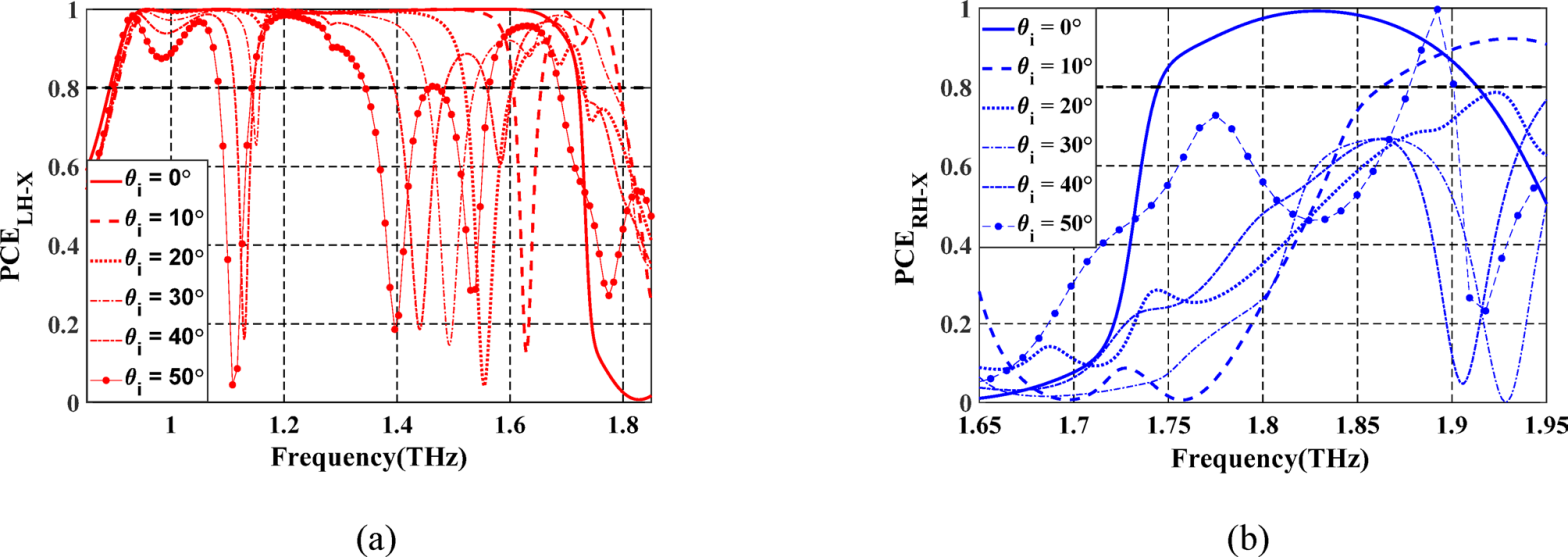
Spectrum of PCE of the proposed LTC-PC under $\:x$-polarized incident wave at different incident angles for (**a**) LH reflected wave. (**b**) RH reflected wave.

To address the angular stability concern, a comprehensive analysis of the PCE is performed under varying incident angles from 0° to 50° for reflection mode as depicted in Fig. 9. For clarity and effective visualization, the results are divided into two spectral sub-bands. The first plot (Fig. 9a) depicts the reaction between 0.85 and 1.85 THz, whereas the second plot (Fig. 9b) concentrates on the 1.65–1.95 THz region. As illustrated in Fig. 9a, the structure maintains consistently high and stable PCE values at incident angles up to 40°, with very modest degradation at 50°. This confirms the design’s good angular robustness and its suitability for practical implementation across a wide angular range. Moreover, based on the geometry of the experimental setup^44^, the maximum practical incidence angle is inherently constrained by the optical path design, which includes the emitter, sample, and receiver alignment. In^44^, the system supports oblique incidence up to approximately 45°, which fully encompasses the angular range considered in our simulation study. Therefore, the angular response characterization presented here adequately reflects the operational boundaries of the measurement system.

Additionally, the frequency bands supporting PCE for the LH-reflected waves at each incident angle are summarized in Table 2. In contrast, as observed from Fig. 9b, the PCE for RH-reflected waves at varying angles remains largely below the 80% threshold, indicating that the designed structure favors efficient LH-polarized reflection under angular variations. A similar angular response was observed when the structure was illuminated with a linearly polarized *y*-oriented incident wave. In this scenario, the polarization conversion behavior remained consistent across various incident angles; nevertheless, the resulting conversion bands are opposite-handed, indicating that the RH-reflected wave is converted to LH and vice versa.

**Table 2:** Frequency bands for the PCE_LH-X_ of the proposed LTC-PC at different incident angles.

$\:{{\varvec{\theta\:}}_{\varvec{i}}}^{\varvec{o}}$	$\:{0}^{\varvec{o}}$	$\:{10}^{\varvec{o}}$	$\:{20}^{\varvec{o}}$	$\:3{0}^{\varvec{o}}$	$\:{40}^{\varvec{o}}$	$\:{50}^{\varvec{o}}$
Frequency bands for PCE_LH−X_	0.90–1.72 THz	0.90–1.60 THz 1.66–1.80 THz	0.90–1.52 THz 1.60–1.73 THz	0.90–1.52 THz 1.60–1.73 THz 1.54–1.78 THz	0.90–1.11 THz 1.14–1.40 THz 1.60–1.73 THz	0.90–1.11 THz 1.14–1.40 THz 1.60–1.73 THz

### Transmission mode with VO_2_ layer in the dielectric state

**Fig. 10:**
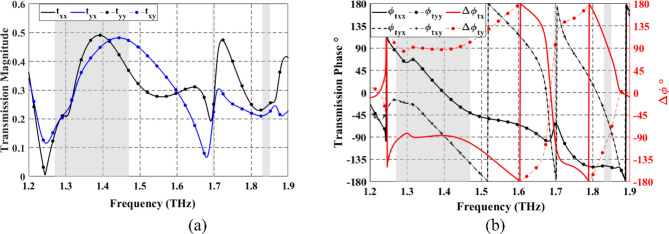
Co- and cross-transmission coefficients from the proposed LTC-PC under $\:x$-polarized and $\:y$-polarized incident waves. (**a**) Transmission magnitude. (**b**) Transmission phase and phase difference. The gray regions depict the frequency ranges for the CP-transmitted waves.

**Fig. 11:**
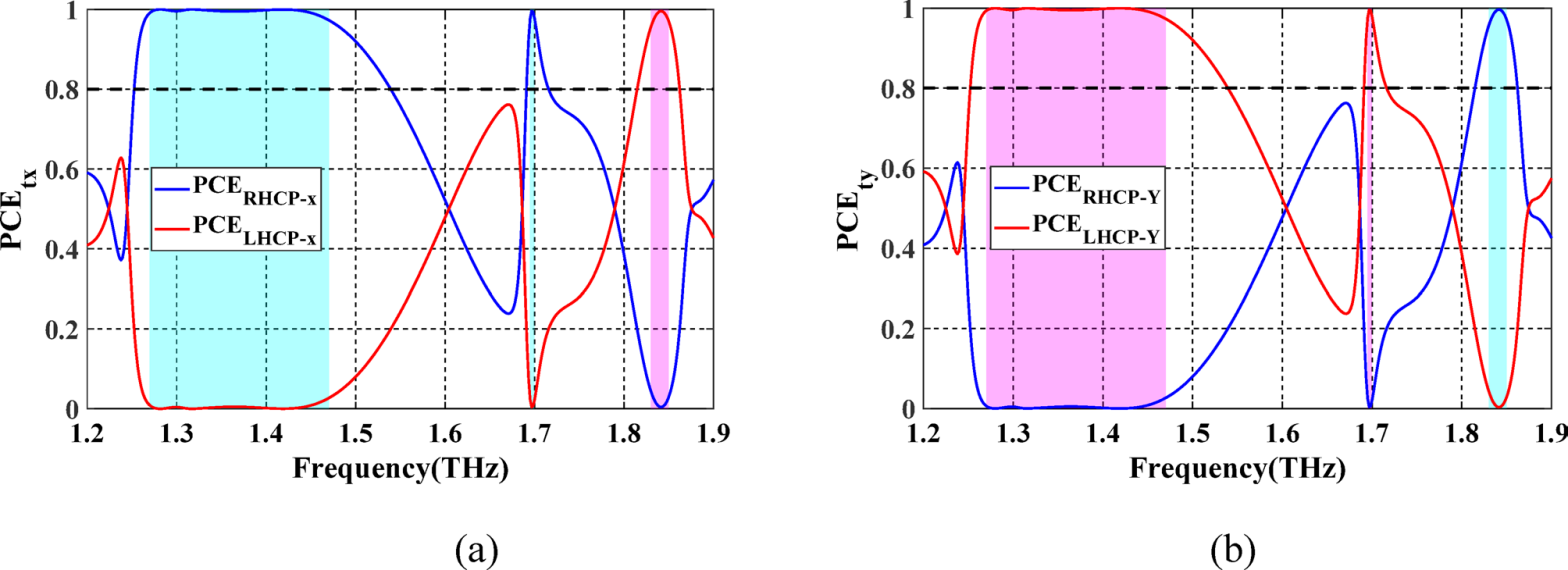
Spectrum of PCE for the proposed LTC-PC under (**a**) $\:x$-polarized incident wave and. (**b**) $\:y$-polarized incident wave. The frequency bands of the LH and RH transmitted waves are shown by the pink and blue sections, respectively.

**Fig. 12:**
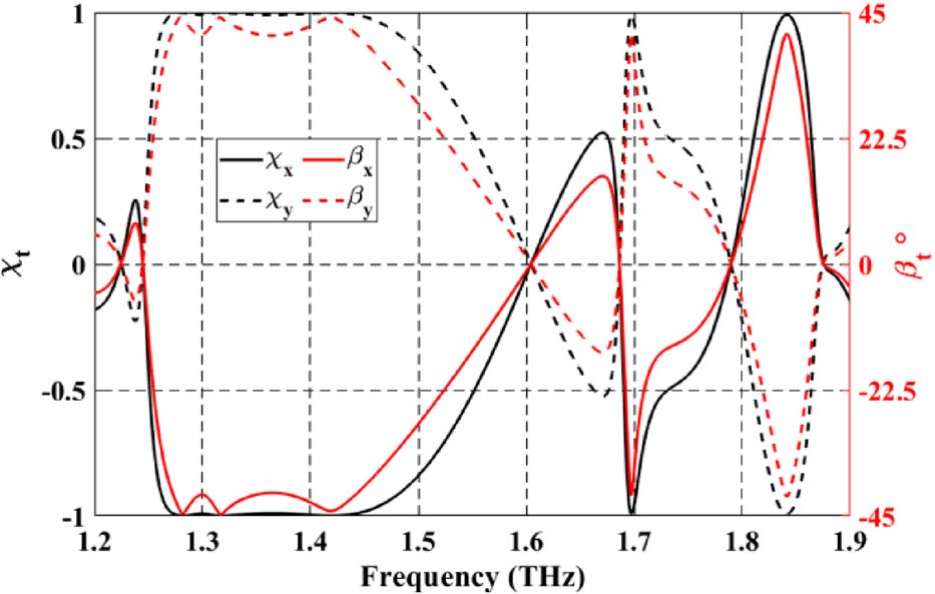
Spectrum of the ellipticity and elliptical angle of the proposed LTC-PC under $\:x$-polarized and $\:y$-polarized incident waves.

When VO_2_ is in the dielectric state, the configuration of top Au pattern-SiO_2_ and bottom Au pattern-SiO_2_ enables the optical transmission of LP incident wave from $\:+z$ to $\:-z$ direction. Figure 10a shows the simulated spectrum for the co- and cross-polarized transmitted magnitudes when illuminated by a linearly *x*- or *y*-polarized incident wave. One can observe that $\:\left({t}_{xx},{t}_{yx}\right)$ and $\:\left({t}_{yy},{t}_{xy}\right)$ are approximately similar in two different frequency bands and at a single frequency. The observed conversion bands are from 1.26 to 1.47 THz and from 1.83 to 1.85 THz, and at a frequency of 1.7 THz with corresponding $\:\varDelta\:{{\upvarphi\:}}_{tx}$ around $\:-90^\circ\:$ and $\:90^\circ\:$, as illustrated in Fig. 10b. Moreover, it can be observed that $\:\varDelta\:{{\upvarphi\:}}_{ty}=-\varDelta\:{{\upvarphi\:}}_{tx}$. The results shown in Fig. 10 satisfy the two conditions required for LTC polarization conversion. The calculated $\:{\text{P}\text{C}\text{E}}_{tx}$ and $\:{\text{P}\text{C}\text{E}}_{ty}$ are demonstrated in Fig. 11a and b, respectively. The calculated ellipticity and ellipticity angle are shown in Fig. 12. In the frequency band from 1.26 to 1.47 THz, the$\:\:{\text{P}\text{C}\text{E}}_{RH-x}\approx\:1$, $\:{\text{P}\text{C}\text{E}}_{LH-x}\approx\:0$, $\:{\beta\:}_{tx}\approx\:-45$° and $\:{\chi\:}_{tx}\approx\:-1$ indicates that the LTC-PC structure converts the LP incident wave into an RH wave with a relative bandwidth of 15.38%. In the frequency range from 1.83 to 1.85 THz, the$\:\:{\text{P}\text{C}\text{E}}_{LH-x}\approx\:1$, $\:{\text{P}\text{C}\text{E}}_{RH-x}\approx\:0$, $\:{\beta\:}_{tx}\approx\:45$° and $\:{\chi\:}_{tx}\approx\:1$ indicates that the transmitted wave is LH. At $\:f$=1.7 THz, $\:{\text{P}\text{C}\text{E}}_{RH-x}$ are greater than 80%, $\:{\beta\:}_{tx}\approx\:45$° and $\:{\chi\:}_{tx}\approx\:0.9$ indicating that the structure converts the incident wave into an RH wave.

**Fig. 13:**
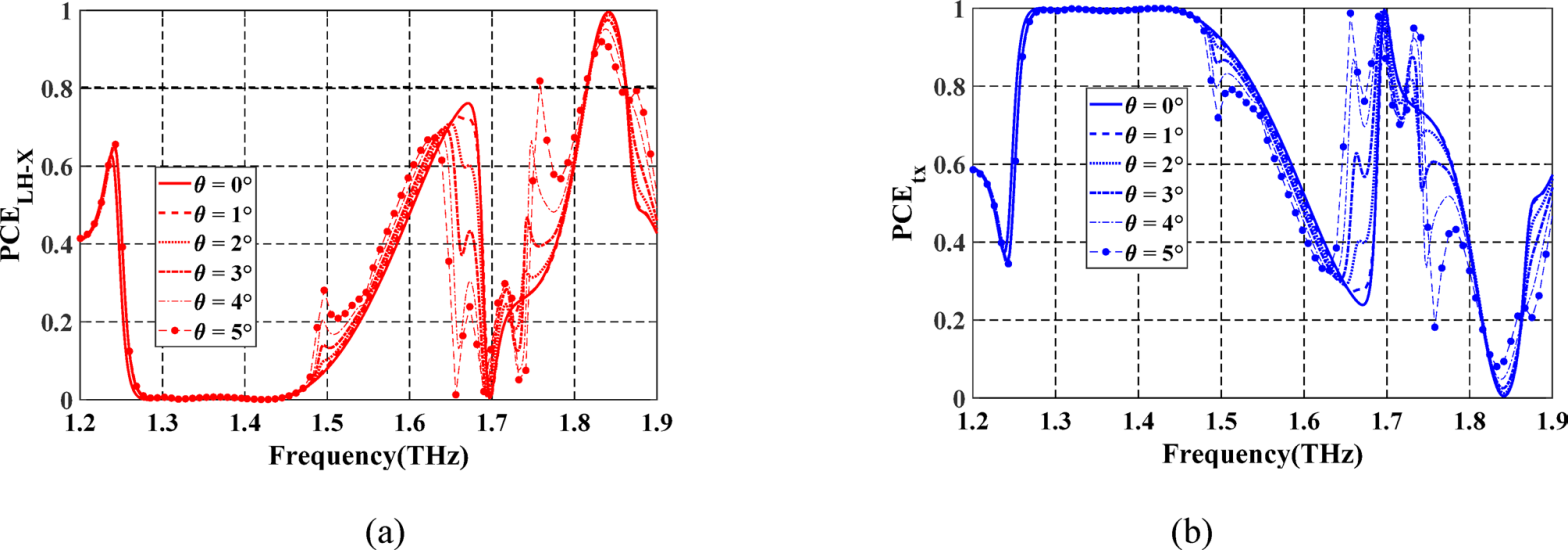
Spectrum of PCE of the proposed LTC-PC under $\:x$-polarized incident wave at different incident angles for (**a**) LH transmitted wave. (**b**) RH transmitted wave.

PCs operating in transmission mode are usually aligned between the THz source and the detector in a normal incidence configuration^20^. In a real experimental setup, especially with fiber-coupled THz systems or collimated optical beams, perfect alignment is difficult^20^. Studying angles up to 5° accounts for minor angular deviations due to misalignment between the source, sample, and detector. To account for practical misalignments in the experimental setup, only a small incident angle variation from 0° to 5° is analyzed, which is sufficient to validate robustness against minor deviations in alignment between the source and the PC. The frequency spectrum of the PCE of the transmitted wave under different incident angles is shown in Fig. 13. It can be observed that the PCE remains stable up to 3^o^ for both RH and LH conversion bands.

### Physical mechanism

Examining the transmission and reflection coefficients along the *u*- and *v*-coordinate systems, respectively, is essential to comprehending the physical mechanisms of polarization conversion in both the transmission and reflection modes. By analyzing how the incident $\:u$-polarized wave reflects or transmits into both $\:u$- and $\:v$-polarized components, and similarly, for $\:v$-polarized incidence, we can directly link the surface current distribution to the generation of these specific polarization states. The relative amplitudes and phases of these polarized components provide direct insights into how the structure manipulates the incident wave to alter its polarization upon reflection or transmission.

**Fig. 14:**
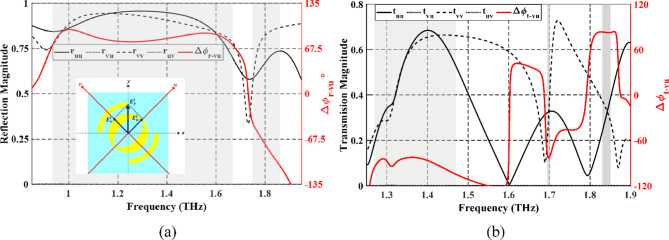
Spectrum of (**a**) reflection coefficients and phase differences and (**b**) transmission coefficients and phase differences, with polarization based on the $\:u$-$\:v$ coordinate system.

Because of the anisotropy of the top Au pattern, the incident $\:x$-polarized wave can be split into two orthogonal components along $\:u$- and $\:v$-coordinate system, which are rotated 45^◦^ around $\:z$ axis with respect to $\:x$- and $\:y$-axes, respectively, as illustrated in the inset of Fig. 14a. The incident wave, in the –*z* direction, is expressed as $\:\stackrel{\rightharpoonup}{{E}_{x}^{i}}=\stackrel{\rightharpoonup}{{E}_{u}^{i}}{+\stackrel{\rightharpoonup}{{E}_{v}^{i}}=E}_{i}{e}^{jkz}\left({\widehat{e}}_{u}+{\widehat{e}}_{v}\right)$. The reflected electric field $\:\stackrel{\rightharpoonup}{{E}_{r}}$ can be decomposed as the orthogonal incident components and expressed as^22^:

20 $$\:\stackrel{\rightharpoonup}{{E}_{r}}={{E}_{i}e}^{jkz}\left[\begin{array}{cc}\begin{array}{c}{r}_{uu}{e}^{{\phi\:}_{r\_uu}}\\\:{r}_{vu{e}^{{\phi\:}_{r\_uv}}}\end{array}&\:\begin{array}{c}{r}_{uv}{e}^{{\phi\:}_{r\_vu}}\\\:{r}_{vv}{e}^{{\phi\:}_{r\_vv}}\end{array}\end{array}\right]\left[\begin{array}{c}{\widehat{e}}_{u}\\\:{\widehat{e}}_{v}\end{array}\right]$$

where $\:\left({r}_{uu},\:{r}_{vu}\right)$ and $\:\left({{r}_{uv},\:r}_{vv}\right)$ are the magnitudes of the reflection coefficients along $\:u$ and $\:v$ directions, respectively, $\:\left({\phi\:}_{r\_uu},\:{\phi\:}_{r\_vu}\right)$ and $\:\left({\phi\:}_{r\_uv},\:{\phi\:}_{r\_vv}\right)$ are the corresponding reflection phases. The phase difference between the two reflection coefficients can be calculated as $\:\varDelta\:{{\upvarphi\:}}_{r-vu}={\phi\:}_{r\_uu}-{\phi\:}_{r\_vv}$.

Also, the transmitted electric field $\:\stackrel{\rightharpoonup}{{E}_{t}}$ can be decomposed as the orthogonal incident components and expressed as^27^:

21 $$\:\stackrel{\rightharpoonup}{{E}_{t}}={{E}_{i}e}^{jkz}\left[\begin{array}{cc}\begin{array}{c}{t}_{uu}{e}^{{\phi\:}_{t\_uu}}\\\:{t}_{vu{e}^{{\phi\:}_{t\_uv}}}\end{array}&\:\begin{array}{c}{t}_{uv}{e}^{{\phi\:}_{t\_vu}}\\\:{t}_{vv}{e}^{{\phi\:}_{t\_vv}}\end{array}\end{array}\right]\left[\begin{array}{c}{\widehat{e}}_{u}\\\:{\widehat{e}}_{v}\end{array}\right]$$

where $\:\left({t}_{uu},\:{t}_{vu}\right)$ and $\:\left({{t}_{uv},\:t}_{vv}\right)$ are the magnitudes of the transmission coefficients along $\:u$ and $\:v$ directions, respectively, $\:\left({\phi\:}_{t\_uu},\:{\phi\:}_{t\_vu}\right)$ and $\:\left({\phi\:}_{t\_uv},\:{\phi\:}_{t\_vv}\right)$ are the corresponding reflection phases. The phase difference between the two transmission coefficients can be calculated as $\:\varDelta\:{{\upvarphi\:}}_{t-vu}={\phi\:}_{r\_uu}-{\phi\:}_{r\_vv}$. When the magnitudes of reflection or transmission coefficients along the $\:u$- and $\:v$-axis directions simultaneously satisfy the relations of $\:{r}_{uu}$ and $\:{r}_{vv}$ are nearly equal and $\:{r}_{vu}\:\approx\:\:{r}_{uv}\approx\:0$ with $\:\varDelta\:{{\upvarphi\:}}_{uv}={\phi\:}_{r\_vv}-{\phi\:}_{{r}_{uu}}\cong\:\pm\:$90°, a circular polarization conversion is achieved, and LH or RH reflected waves can be obtained^22^.

When the LTC-PC structure works in reflection mode, it can be observed that $\:{r}_{uu}$ and $\:{r}_{vv}$ are nearly equal and greater than 0.8 in the frequency range of 0.93–1.67 THz and 1.80–1.86 THz, as shown in Fig. 14a, while $\:{r}_{vu}$ and $\:{r}_{uv}$ are very small and can almost be ignored. On the other hand, $\:\varDelta\:{{\upvarphi\:}}_{vu}\approx\:$90° and $\:-$90°, meaning that the reflected waves are LH and RH in the two operating bands, respectively. Similarly, when the structure works in transmission mode, $\:{t}_{uu}$ and $\:{t}_{vv}$ are nearly equal and $\:\varDelta\:{{\upvarphi\:}}_{t-vu}$ is close to $\:-$90° in the frequency range of 1.26 to 1.47 THz and at 1.7 THz, as shown in Fig. 14b. Moreover, $\:{t}_{uu}$ and $\:{t}_{vv}$ are similar with a phase difference close to 90° in the frequency range of 1.83 to 1.85 THz, indicating the LP incident wave converted into LH.

**Fig. 15:**
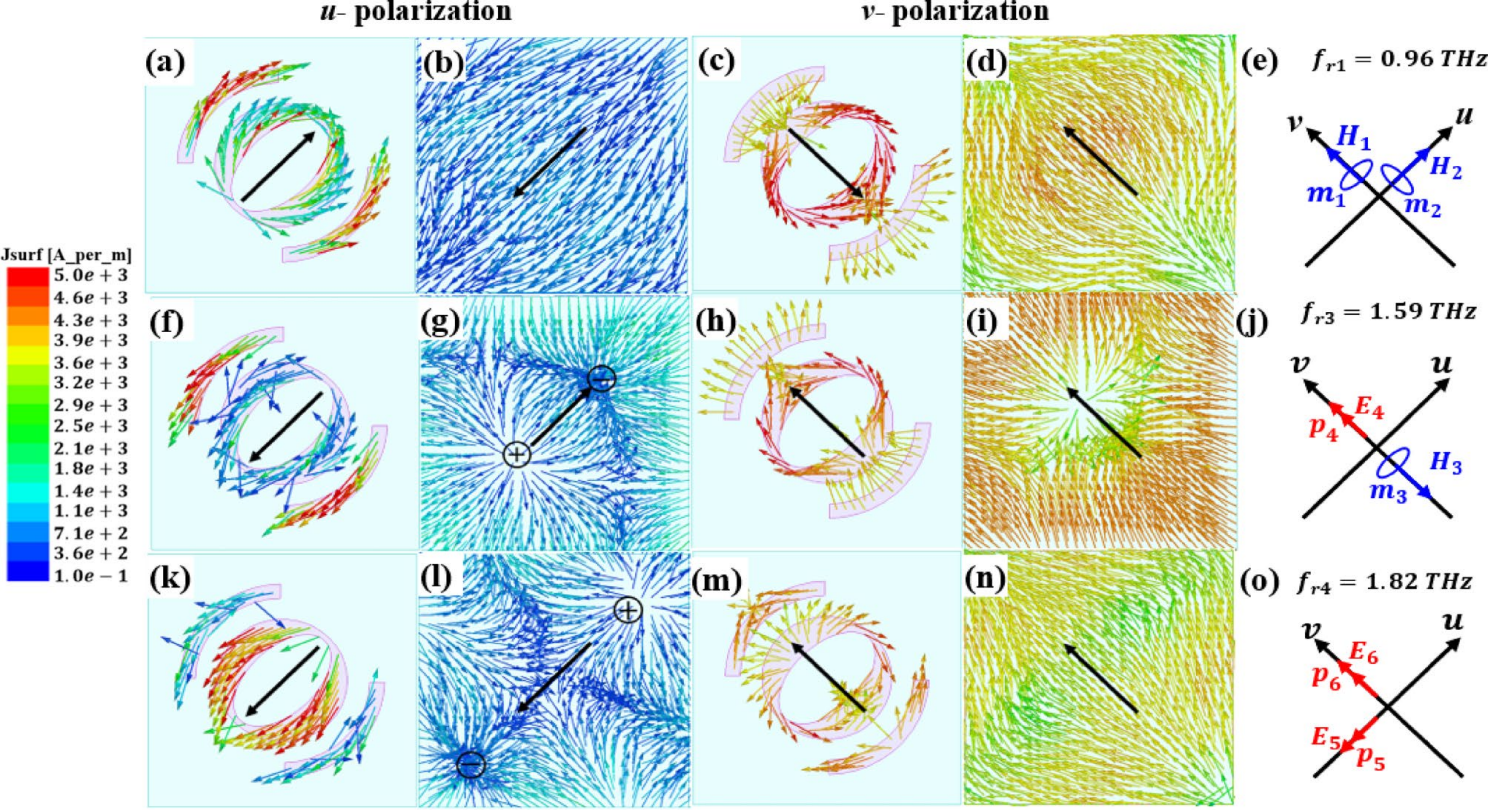
Surface current distributions and the corresponding electric and magnetic moments in the reflection mode.

The current distributions of the top Au pattern and the VO_2_ layer in the *u*-*v* coordinate system now enhance our understanding of how polarization conversion functions in reflection mode. Three frequencies of nearly-perfect $\:\text{P}\text{C}\text{E}$ and AR$\:\ll\:$3 dB are selected to analyze the surface current, which are 0.96 and 1.59 THz for LH and 1.82 THz for RH, as indicated in Fig. 15. It’s important to note that the surface current distribution was determined using the HFSS simulator. In Fig. 15, the first and third columns show the current distribution on the top Au pattern, and the second and fourth columns show the current distribution on the VO_2_ layer in the metallic state. The first and second columns are for reflection under *u*-polarized incident waves. The surface current distribution under $\:v$-polarized incident waves is shown in the third and fourth columns. The equivalent magnetic moment (circled in blue) and electric dipole moment (red double-headed arrows) are plotted in the fifth column. For ease of viewing, a thick black arrow indicates the general direction of the current. In general, if the current on the top Au pattern is parallel to the current on the metallic VO_2_ layer, electric resonance will be generated. Conversely, current loops in the dielectric substrate will create magnetic resonance if the surface current on the top pattern is antiparallel to the current on the metallic VO_2_ layer. At $\:{f}_{r1}$=0.96 THz as shown in Fig. 15a and c, the current on the top Au is antiparallel to that on the metallic VO_2_ (Fig. 15b and d) under both $\:u$- and $\:v$-polarizations, meaning that equivalent magnetic moments $\:{m}_{1}$ and $\:{m}_{2}$ are produced as shown in Fig. 15e. These magnetic resonances control the magnitudes and phases of the reflected electric fields along the *u*- and *v*-axis, which are almost equal in magnitude with a phase difference of + 90° resulting in an LH wave. From Fig. 15f and g, at $\:{f}_{r2}$ = 1.59 THz, an equivalent magnetic moment *m*_3_ is produced in the -*v* direction by the antiparallel surface currents at the top layer and the reflection layer, which form a closed loop to induce magnetic resonance. Under $\:v$-polarization, the surface currents on the top and reflection layers are in the same direction as shown in Fig. 15h and i, which induces an electric resonance and produces an equivalent electrical dipole moment $\:{\text{p}}_{4}$ in the $\:+v$ direction. Both $\:{m}_{3}$ and $\:{\text{p}}_{4}$ simultaneously manipulating the reflection magnitudes with a phase difference of +90° resulting in an LH as shown in Fig. 15j. Finally, at $\:{f}_{r3}$ = 1.82 THz as shown in Fig. 15k, l, m and n, and 15o, two equivalent electric dipole moments $\:{\text{p}}_{5}$ and $\:{\text{p}}_{6}$ are produced under both $\:u$ and $\:v$-polarizations, respectively, manipulating the reflection magnitudes with a phase difference of $\:-90^\circ\:$ resulting in an RH wave.

**Fig. 16:**
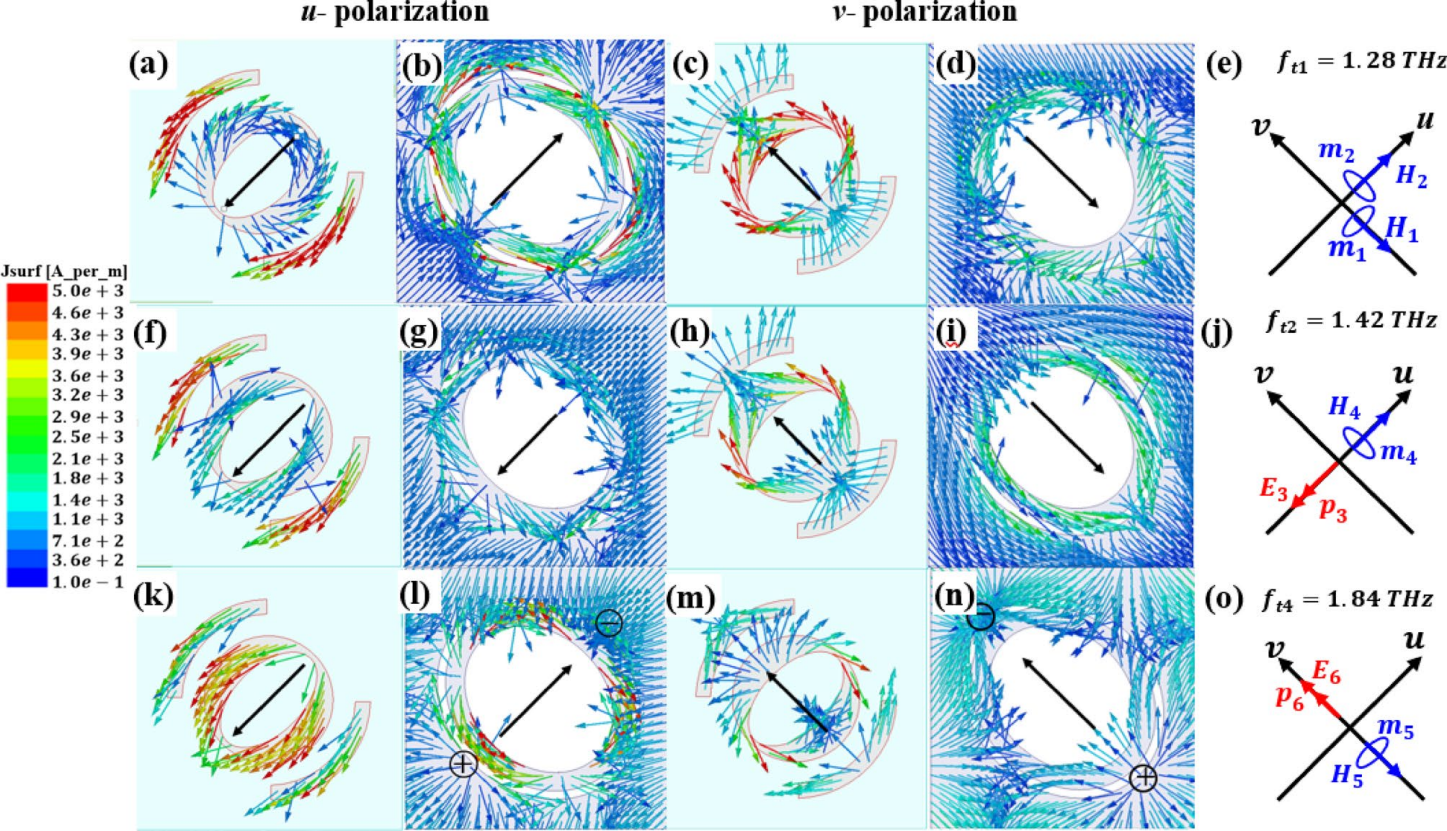
Surface current distributions and the corresponding electric and magnetic moments in the transmission mode.

When the LTC-PC structure works in the transmission mode, VO_2_ behaves as an insulator. In this case, the surface currents are distributed on the top Au pattern and the back Au pattern. Three resonance frequencies are selected to study the surface current in this mode: 1.28, 1.42, and 1.84 THz. At $\:{f}_{t1}$=1.28 THz as shown in Fig. 16a and c, the current on the top Au is antiparallel to that on the back Au pattern (Fig. 16b and d) under both $\:u$ and $\:v$ polarizations, meaning that two orthogonal magnetic moments $\:{m}_{1}$ and $\:{m}_{2}$ are produced as shown in Fig. 16e. These magnetic resonances control the magnitudes and phases of the transmitted electric fields along the *u*- and *v*-axis, which are approximately equal in magnitude with a phase difference of $\:-$90° resulting in an RH wave. At $\:{f}_{t2}$ = 1.42 THz, under $\:u$-polarization, the surface current distributions on the top and back Au layers are in the same direction as shown in Fig. 16f and g, which induce an electric resonance and produce an electric dipole moment $\:{\text{p}}_{3}$ in the $\:-u$ direction. Under $\:v$-polarization, the surface currents are antiparallel on the two layers as shown in Fig. 16h and i. This forms an equivalent magnetic moment $\:{m}_{4}$ in the $\:+u$ direction. Both $\:{\text{p}}_{3}$ and $\:{m}_{4}$ simultaneously manipulating the transmission magnitudes with a phase difference of $\:-$90° resulting in an RH as shown in Fig. 16j. Finally, at $\:{f}_{t3}$ = 1.84 THz, the surface current distributions are illustrated in Fig. 16k, l and m, and 16n. Magnetic moment $\:{m}_{5}$ and electric dipole moment $\:{\text{p}}_{6}$ are produced under both $\:u$ and $\:v$-polarizations, respectively, manipulating the transmission magnitudes with a phase difference of $\:90^\circ\:$ resulting in an LH wave, as shown in Fig. 16o.

### Comparison study

This section compares the proposed dual-mode LTC-PC structure to the recently proposed dual-mode PCs. The comparison illustrated in Table 3 is based on the working modes, the functionality of the PC in each mode (i.e., LTL or LTC), the frequency bands for each mode, the basic materials that are responsible for the dual functionality, and the dual mode control method (thermal/optical).

## Conclusions

In the presented paper, we reported an LTC dual-mode PC. The switching between LTC conversion in transmission or reflection modes is based on controlling the state of the phase-change material VO_₂_. When VO_2_ is in a dielectric state, the proposed converter works in transmission mode. In this mode, the LP incident THz waves interact with the top and bottom Au patterns and dielectric material, and hence are converted into CP transmitted THz waves. Under the given structure parameters, the simulation study indicated an LTC polarization conversion in the frequency ranges of 1.26 to 1.47 THz and 1.83 to 1.85 THz. Moreover, this mode yields an LTC polarization conversion at a frequency of 1.7 THz. When VO_2_ is in a metallic state, the proposed converter works in reflection mode. In this mode, the LP incident THz waves interact with the top Au pattern, dielectric layer, and the VO₂ metal reflector; hence, they are converted into CP reflected THz waves. Two conversion bands are identified for circular polarization within the frequency range of 0.93–1.67 THz and 1.80–1.86 THz. The dual-mode polarization converter achieves polarization conversion efficiencies exceeding 0.8 and axial ratios below 3 dB in both transmission and reflection modes. Through theoretical analysis and surface current distributions, we have comprehensively examined the fundamental mechanisms for both transmission and reflection modes. Due to its dual-mode functioning, this suggested metamaterial shows great promise for a variety of uses, including THz communication, sensing, and imaging.

**Table 3:** Comparison between the recently reported dual-mode PC and the proposed dual-mode LTC-PC.

References	Working modes	Functionality	Frequency bands	Basic materials	Dual-mode control
^24^	Transmission Reflection	LTL LTL	7.5–10.8 THz 6.1–13.3 THz	VO_2_	Thermal
^25^	Transmission Reflection	LTL LTL	0.52–1.74 THz 0.62–1.70 THz	VO_2_	Thermal
^26^	Transmission Reflection	LTL LTL	0.75–4.0 THz 1.4–3.3 THz	VO_2_	Thermal
^27^	Transmission Reflection	LTL LTL	0.40–1.24 THz 0.44–1.30 THz	Ge and Si	Optical
^28^	Transmission Reflection	LTL LTL and LTC	0.32–3.44 THz 1.19–3.48 THz, 1.61–3.38 THz, 1.24–1.29 THz	VO_2_ and Si	Thermal/optical
^29^	Transmission Reflection	LTL LTL	3.00–6.00 THz 3.76–4.15 THz	VO_2_	Thermal
**This work**	Transmission Reflection	LTC LTC	1.26 − 1.47 THz, 1.83 − 1.85 THz, 1.7 THz 0.93–1.67 THz, 1.80–1.86 THz	VO_2_	Thermal

## Data Availability

The datasets used and/or analysed during the current study available from the corresponding author on reasonable request.
